# Meta-analyses of Adverse Effects Data Derived from Randomised Controlled Trials as Compared to Observational Studies: Methodological Overview

**DOI:** 10.1371/journal.pmed.1001026

**Published:** 2011-05-03

**Authors:** Su Golder, Yoon K. Loke, Martin Bland

**Affiliations:** 1 Centre for Reviews and Dissemination, University of York, York, United Kingdom; 2 School of Medicine, University of East Anglia, Norwich, United Kingdom; 3 Department of Health Sciences, University of York, York, United Kingdom; Leiden University Hospital, The Netherlands

## Abstract

**Background:**

There is considerable debate as to the relative merits of using randomised controlled trial (RCT) data as opposed to observational data in systematic reviews of adverse effects. This meta-analysis of meta-analyses aimed to assess the level of agreement or disagreement in the estimates of harm derived from meta-analysis of RCTs as compared to meta-analysis of observational studies.

**Methods and Findings:**

Searches were carried out in ten databases in addition to reference checking, contacting experts, citation searches, and hand-searching key journals, conference proceedings, and Web sites. Studies were included where a pooled relative measure of an adverse effect (odds ratio or risk ratio) from RCTs could be directly compared, using the ratio of odds ratios, with the pooled estimate for the same adverse effect arising from observational studies. Nineteen studies, yielding 58 meta-analyses, were identified for inclusion. The pooled ratio of odds ratios of RCTs compared to observational studies was estimated to be 1.03 (95% confidence interval 0.93–1.15). There was less discrepancy with larger studies. The symmetric funnel plot suggests that there is no consistent difference between risk estimates from meta-analysis of RCT data and those from meta-analysis of observational studies. In almost all instances, the estimates of harm from meta-analyses of the different study designs had 95% confidence intervals that overlapped (54/58, 93%). In terms of statistical significance, in nearly two-thirds (37/58, 64%), the results agreed (both studies showing a significant increase or significant decrease or both showing no significant difference). In only one meta-analysis about one adverse effect was there opposing statistical significance.

**Conclusions:**

Empirical evidence from this overview indicates that there is no difference on average in the risk estimate of adverse effects of an intervention derived from meta-analyses of RCTs and meta-analyses of observational studies. This suggests that systematic reviews of adverse effects should not be restricted to specific study types.

*Please see later in the article for the Editors' Summary*

## Abstract 2

Su Golder and colleagues carry out an overview of meta-analyses to assess whether estimates of the risk of harm outcomes differ between randomized trials and observational studies. They find that, on average, there is no difference in the estimates of risk between overviews of observational studies and overviews of randomized trials.

## Editors' Summary

**Background:**

Whenever patients consult a doctor, they expect the treatments they receive to be effective and to have minimal adverse effects (side effects). To ensure that this is the case, all treatments now undergo exhaustive clinical research—carefully designed investigations that test new treatments and therapies in people. Clinical investigations fall into two main groups—randomized controlled trials (RCTs) and observational, or non-randomized, studies. In RCTs, groups of patients with a specific disease or condition are randomly assigned to receive the new treatment or a control treatment, and the outcomes (for example, improvements in health and the occurrence of specific adverse effects) of the two groups of patients are compared. Because the patients are randomly chosen, differences in outcomes between the two groups are likely to be treatment-related. In observational studies, patients who are receiving a specific treatment are enrolled and outcomes in this group are compared to those in a similar group of untreated patients. Because the patient groups are not randomly chosen, differences in outcomes between cases and controls may be the result of a hidden shared characteristic among the cases rather than treatment-related (so-called confounding variables).

**Why Was This Study Done?:**

Although data from individual trials and studies are valuable, much more information about a potential new treatment can be obtained by systematically reviewing all the evidence and then doing a meta-analysis (so-called evidence-based medicine). A systematic review uses predefined criteria to identify all the research on a treatment; meta-analysis is a statistical method for combining the results of several studies to yield “pooled estimates” of the treatment effect (the efficacy of a treatment) and the risk of harm. Treatment effect estimates can differ between RCTs and observational studies, but what about adverse effect estimates? Can different study designs provide a consistent picture of the risk of harm, or are the results from different study designs so disparate that it would be meaningless to combine them in a single review? In this methodological overview, which comprises a systematic review and meta-analyses, the researchers assess the level of agreement in the estimates of harm derived from meta-analysis of RCTs with estimates derived from meta-analysis of observational studies.

**What Did the Researchers Do and Find?:**

The researchers searched literature databases and reference lists, consulted experts, and hand-searched various other sources for studies in which the pooled estimate of an adverse effect from RCTs could be directly compared to the pooled estimate for the same adverse effect from observational studies. They identified 19 studies that together covered 58 separate adverse effects. In almost all instances, the estimates of harm obtained from meta-analyses of RCTs and observational studies had overlapping 95% confidence intervals. That is, in statistical terms, the estimates of harm were similar. Moreover, in nearly two-thirds of cases, there was agreement between RCTs and observational studies about whether a treatment caused a significant increase in adverse effects, a significant decrease, or no significant change (a significant change is one unlikely to have occurred by chance). Finally, the researchers used meta-analysis to calculate that the pooled ratio of the odds ratios (a statistical measurement of risk) of RCTs compared to observational studies was 1.03. This figure suggests that there was no consistent difference between risk estimates obtained from meta-analysis of RCT data and those obtained from meta-analysis of observational study data.

**What Do These Findings Mean?:**

The findings of this methodological overview suggest that there is no difference on average in the risk estimate of an intervention's adverse effects obtained from meta-analyses of RCTs and from meta-analyses of observational studies. Although limited by some aspects of its design, this overview has several important implications for the conduct of systematic reviews of adverse effects. In particular, it suggests that, rather than limiting systematic reviews to certain study designs, it might be better to evaluate a broad range of studies. In this way, it might be possible to build a more complete, more generalizable picture of potential harms associated with an intervention, without any loss of validity, than by evaluating a single type of study. Such a picture, in combination with estimates of treatment effects also obtained from systematic reviews and meta-analyses, would help clinicians decide the best treatment for their patients.

**Additional Information:**

Please access these Web sites via the online version of this summary at http://dx.doi.org/10.1371/journal.pmed.1001026.

- The US National Institutes of Health provide information on clinical research; the UK National Health Service Choices Web site also has a page on clinical trials and medical research
- The Cochrane Collaboration produces and disseminates systematic reviews of health-care interventions
- Medline Plus provides links to further information about clinical trials (in English and Spanish)

## Introduction

There is considerable debate regarding the relative utility of different study designs in generating reliable quantitative estimates for the risk of adverse effects. A diverse range of study designs encompassing randomised controlled trials (RCTs) and non-randomised studies (such as cohort or case-control studies) may potentially record adverse effects of interventions and provide useful data for systematic reviews and meta-analyses [1],[2]. However, there are strengths and weaknesses inherent to each study design, and different estimates and inferences about adverse effects may arise depending on study type [3].

In theory, well-conducted RCTs yield unbiased estimates of treatment effect, but there is often a distinct lack of RCT data on adverse effects [2],[4]–[7]. It is often impractical, too expensive, or ethically difficult to investigate rare, long-term adverse effects with RCTs [5],[7]–[16]. Empirical studies have shown that many RCTs fail to provide detailed adverse effects data, that the quality of those that do report adverse effects is poor [6],[17]–[31], and that the reporting may be strongly influenced by expectations of investigators and patients [32].

In general RCTs are designed and powered to explore efficacy [1],[3],[9],[30],[33]. As the intended effects of treatment are more likely to occur than adverse effects and to occur within the trial time frame, RCTs may not be large enough, or have a sufficient follow-up to identify rare, long-term adverse effects, or adverse effects that occur after the drug has been discontinued [1]–[3],[9],[13],[15],[16],[18],[19],[21],[26],[30],[33]–[53]. Moreover, generalisability of RCT data may be limited if, as is often the case, trials specifically exclude patients at high risk of adverse effects, such as children, the elderly, pregnant women, patients with multiple comorbidities, and those with potential drug interactions [1]–[3],[15],[38],[39],[41],[45],[46],[54]–[56].

Given these limitations it may be important to evaluate the use of data from non-randomised studies in systematic reviews of adverse effects. Owing to the lack of randomisation, all types of observational studies are potentially afflicted by an increased risk of bias (particularly from confounding) [8],[57] and may therefore be a much weaker study design for establishing causation [12]. Nevertheless, observational study designs may sometimes be the only available source of data for a particular adverse effect, and are commonly used in evaluating adverse effects [1],[9],[13],[52],[58],[59]. It is also debatable how important it is to control for confounding by indication for unanticipated adverse effects. Authors have argued that confounding is less likely to occur when an outcome is unintended or unanticipated than when the outcome is an intended effect of the exposure. This is because the potential for that adverse effect is not usually associated with the reasons for choosing a particular treatment, and therefore does not influence the prescribing decision [52],[59]–[62]. For instance, in considering the risk of venous thrombosis from oral contraceptives in healthy young women, the choice of contraceptive may not be linked to risk factors for deep venous thrombosis (an adverse effect that is not anticipated). Thus, any difference in rates of venous thrombosis may be due to a difference in the risk of harm between contraceptives [52],[62].

As both RCTs and observational studies are potentially valuable sources of adverse effects data for meta-analysis, the extent of any discrepancy between the pooled risk estimates from different study designs is a key concern for systematic reviewers. Previous research has tended to focus on differences in treatment effect between RCTs and observational studies [63]–[69]. However, estimates of beneficial effects may potentially be prone to different biases to estimates of adverse effects amongst the different study designs. Can the different study designs provide a consistent picture on the risk of harm, or are the results from different study designs so disparate that it would not be meaningful to combine them in a single review? This uncertainty has not been fully addressed in current methodological guidance on systematic reviews of harms [46], probably because the existing research has so far been inconclusive, with examples of both agreement and disagreement in the reported risk of adverse effects between RCTs and observational studies [1],[11],[15],[48],[51],[70]–[78]. In this meta-analysis of meta-analyses, we aimed to compare the estimates of harm (for specific adverse effects) reported in meta-analysis of RCTs with those reported in meta-analysis of observational studies for the same adverse effect.

## Methods

### Search Strategy

Broad, non-specific searches were undertaken in ten electronic databases to retrieve methodology papers related to any aspect of the incorporation of adverse effects into systematic reviews. A list of the databases and other sources searched is given in Text S1. In addition, the bibliographies of any eligible articles identified were checked for additional references, and citation searches were carried out for all included references using ISI Web of Knowledge. The search strategy used to identify relevant methodological studies in the Cochrane Methodology Register is described in full in Text S2. This strategy was translated as appropriate for the other databases. No language restrictions were applied to the search strategies. However, because of logistical constraints, only non-English papers for which a translation was readily available were retrieved.

Because of the limitations of searching for methodological papers, it was envisaged that relevant papers may be missed by searching databases alone. We therefore undertook hand-searching of selected key journals, conference proceedings, and Web sources, and made contact with other researchers in the field. In particular, one reviewer (S. G.) undertook a detailed hand search focusing on the Cochrane Database of Systematic Reviews and the Database of Abstracts of Reviews of Effects (DARE) to identify systematic reviews that had evaluated adverse effects as a primary outcome. A second reviewer (Y. K. L.) checked the included and excluded papers that arose from this hand search.

### Inclusion Criteria

A meta-analysis or evaluation study was considered eligible for inclusion in this review if it evaluated studies of more than one type of design (for example, RCTs versus cohort or case-control studies) on the identification and/or quantification of adverse effects of health-care interventions. We were principally interested in meta-analyses that reported pooled estimates of the risk of adverse effects according to study designs that the authors stated as RCTs, as opposed to analytic epidemiologic studies such as case-control and controlled cohort studies (which authors may have lumped together as a single “observational” category). Our review focuses on the meta-analyses where it was possible to compare the pooled risk ratios (RRs) or odds ratios (ORs) from RCTs against those from other study designs.

### Data Extraction

Information was collected on the primary objective of the meta-analyses; the adverse effects, study designs, and interventions included; the number of included studies and number of patients by study design; the number of adverse effects in the treatment and control arm or comparator group; and the type of outcome statistic used in evaluating risk of harm.

We relied on the categorisation of study design as specified by the authors of the meta-analysis. For example, if the author stated that they compared RCTs with cohort studies, we assumed that the studies were indeed RCTs and cohort studies.

Validity assessment and data extraction were carried out by one reviewer (S. G.), and checked by a second reviewer (Y. K. L.). All discrepancies were resolved after going back to the original source papers, with full consensus reached after discussion.

### Validity Assessment

The following criteria were used to consider the validity of comparing risk estimates across different study designs. (1) Presence of confounding factors: Discrepancies between the results of RCTs and observational studies may arise because of factors (e.g., differences in population, administration of intervention, or outcome definition) other than study design. We recorded whether the authors of the meta-analysis checked if the RCTs and observational studies shared similar features in terms of population, interventions, comparators, and measurement of outcomes and whether they used methods such as restriction or stratification by population, intervention, comparators, or outcomes to improve the comparability of pooled risk estimates arising from different groups of studies. (2) Heterogeneity by study design: We recorded whether the authors of the meta-analysis explored heterogeneity of the pooled studies by study design (using measures such as Chi^2^ or *I* ^2^). We assessed the extent of heterogeneity of each meta-analysis using a cut-off point of *p* < 0.10 for Chi^2^ test results, and we specifically looked for instances where *I* ^2^ was reported as above 50%. In the few instances where both statistics were presented, the results of *I* ^2^ were given precedence [79]. (3) Statistical analysis comparing study designs: We recorded whether the authors of the meta-analysis described the statistical methods by which the magnitude of the difference between study designs was assessed.

### Data Analysis

A descriptive summary of the data in terms of confidence interval (CI) overlap between pooled sets of results by study design, and any differences in the direction of effect between study designs, were presented. The results were said to agree if both study designs identified a significant increase, a significant decrease, or no significant difference in the adverse effects under investigation.

Quantitative differences or discrepancies between the pooled estimates from the respective study designs for each adverse effect were illustrated by taking the ratio of odds ratios (ROR) from meta-analysis of RCTs versus meta-analysis of observational studies. We calculated ROR by using the pooled OR for the adverse outcome from RCTs divided by the pooled OR for the adverse outcome from observational studies. If the meta-analysis of RCTs for a particular adverse effect yielded exactly the same OR as the meta-analysis of observational studies (i.e., complete agreement, or no discrepancy between study designs), then the ROR would be 1.0 (and ln ROR = 0). Because adverse events are rare, ORs and RRs were treated as equivalent [80].

The estimated ROR from each “RCT versus observational study” comparison was then used in a meta-analysis (random effects inverse variance method; RevMan 5.0.25) to summarize the overall ROR between RCTs and observational studies across all the included reviews. The standard error (SE) of ROR can be estimated using the SEs for the RCT and observational estimates:
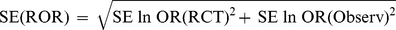
(1)


SEs pertaining to each pooled OR(RCT) and OR(Observ) were calculated from the published 95% CI.

Statistical heterogeneity was assessed using *I* ^2^ statistic, with *I* ^2^ values of 30%–60% representing a moderate level of heterogeneity [81].

## Results

### Included Studies

In total, 52 articles were identified as potentially eligible for this review. On further detailed evaluation, 33 of these articles either compared different types of observational studies to one another (for example, cohort studies versus case control studies) or compared only the incidence of adverse effects (without reporting the RR/OR) in those receiving the intervention according to type of study [57],[82]–[113].

**Figure 1:**
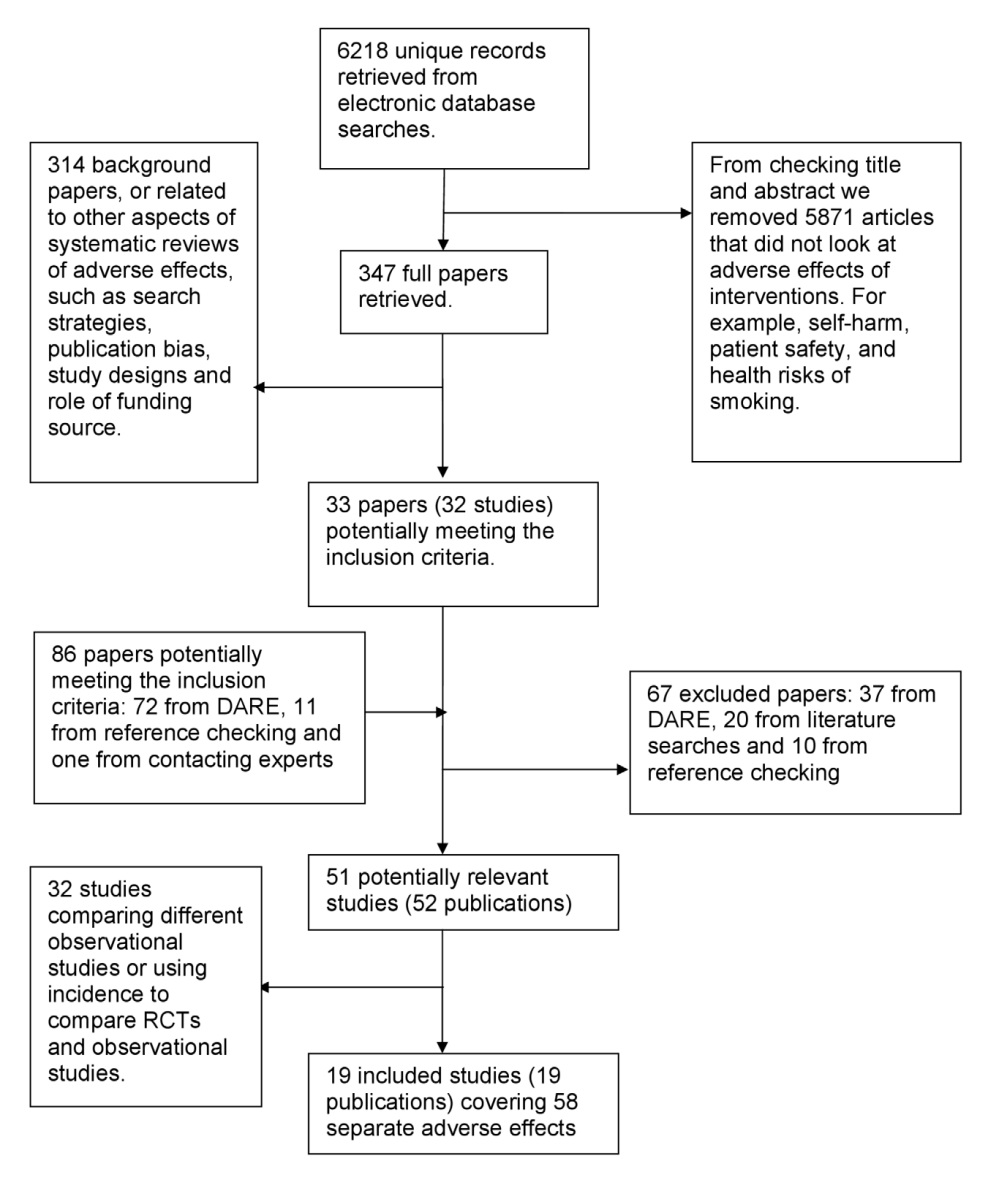
Flow chart for included studies.

We finally selected 19 eligible articles that compared the relative risk or ORs from RCTs and observational studies (Figure 1) [6],[114]–[131]. These 19 articles covering meta-analysis of 58 separate adverse effects will be the focus of this paper. The 58 meta-analyses included a total of over 311 RCTs and over 222 observational studies (comprising 57 cohort studies, 75 case-control studies, and at least 90 studies described as “observational” by the authors without specifying the exact type) (Table S1). (Exact numbers of RCTs and observational studies cannot be calculated as overlap in the included studies in McGettigan and Henry [127] could not be ascertained.)

Two of the 19 articles were methodological evaluations with the main aim of assessing the influence of study characteristics (including study design) on the measurement of adverse effects [6],[127], whereas the remaining 17 were systematic reviews within which subgroup analysis by study design was embedded [114]–[126],[128]–[131] (Table S1).

### Adverse Effects

The majority of the articles compared the results from RCTs and observational studies using only one adverse effect (11/19, 58%) [114],[115],[117]–[119],[121],[122],[124],[125],[129],[130], whilst three included one type of adverse effect (such as cancer, gastrointestinal complications, or cardiovascular events) [116],[127],[128], and five articles included a number of specified adverse effects (ranging from two to nine effects) or any adverse effects [6],[120],[123],[126],[131].

### Interventions

Most (17/19, 89%) of the articles included only one type of intervention (such as hormone replacement therapy [HRT] or nonsteroidal anti-inflammatory drugs) [114]–[120],[122]–[131], whilst one article looked at two interventions (HRT and oral contraceptives) [121] and another included nine interventions [6]. Most of the analyses focused on the adverse effects of pharmacological interventions; however, other topics assessed were surgical interventions (such as bone marrow transplantation and hernia operations) [6],[120] and a diagnostic test (ultrasonography) [131].

### Excluded Studies

Text S3 lists the 67 studies that were excluded from this systematic review during the screening and data extraction phases, with the reasons for exclusion.

### Summary of Methodological Quality

#### Role of confounding factors

Although many of the meta-analyses acknowledged the potential for confounding factors that might yield discrepant findings between study designs, no adjustment for confounding factors was reported in most instances [6],[114]–[116],[118]–[122],[124]–[126],[128]–[131]. However, a few authors did carry out subgroup analysis stratified for factors such as population characteristics, drug dose, or duration of drug exposure.

There were two instances where the authors of the meta-analysis performed some adjustment for potential confounding factors: one carried out meta-regression [123], and in the other methodological evaluation the adjustment method carried out was unclear [127].

#### Heterogeneity by study design

Thirteen meta-analyses measured the heterogeneity of at least one set of the included studies grouped by study design using statistical analysis such as Chi^2^ or *I* ^2^ [6],[115]–[117],[119],[121],[123]–[125],[127],[129]–[131].

The pooled sets of RCTs were least likely to exhibit any strong indication of heterogeneity; only five (15%) [6],[117],[130],[131] of the 33 [6],[115]–[117],[119],[121],[123],[124],[129]–[131] sets of pooled RCTs were significantly heterogeneous, and in two of these sets of RCTs the heterogeneity was only moderate, with *I* ^2^ *= *58.9% [117] and *I* ^2^ *= *58.8% [130].

Three of the four case-control studies, one of the four cohort studies, and 14 of the 25 studies described as “observational studies” also exhibited substantial heterogeneity.

#### Statistical analysis comparing study designs

Authors of one meta-analysis explicitly tested for a difference between the results of the different study designs [6]. Two other analyses reported on the heterogeneity of the pooled RCTs, the pooled observational studies, and the pooled RCTs and observational studies, which can indicate statistical differences where the pooled study designs combined are significantly heterogenous but no significant heterogeneity is seen when the study designs are pooled separately.

### Data Analysis

Text S4 documents the decisions made in instances where the same data were available in more than one format.

#### Size of studies

In ten methodological evaluations the total number of participants was reported in each set of pooled studies by study design [114],[116],[118]–[120],[123],[125],[126],[130],[131], and in another five methodological evaluations the pooled number of participants was reported for at least one type of study design [6],[124],[127]–[129]. Studies described as “observational” by the authors contained the highest number of participants per study, 34,529 (3,798,154 participants/110 studies), followed by cohort studies, 33,613 (1,378,131 participants/41 studies). RCTs and case-control studies had fewer participants, 2,228 (821,954 participants/369 studies) and 2,144 (105,067 participants/49 studies), respectively.

#### Confidence interval overlap

In almost all instances the CIs for the pooled results from the different study designs overlapped (Table 1). However, there were four pooled sets of results in three methodological evaluations where the CIs did not overlap [6],[119],[121].

**Table 1:** Confidence interval overlap and agreement between study designs.

Study Design Comparisons	CIs Overlapped	Agreement in Findings between the Study Designs				Discrepancy in Findings between the Study Designs			
		Both Showed a Significant Increase	Both Did Not Identify Any Significant Difference	Both Showed a Significant Decrease	Total for Any Agreement	Significant Risk Increase in One vs. Significant Risk Decrease in the Other	Significant Increase In One vs. No Significant Difference in the Other	Significant Decrease in One vs. No Difference in the Other	Total for Any Disagreement
RCTs vs. all “observational” studies (*n = *58)	54 (93%)	11 (19%)	23 (40%)	3 (5%)	37 (64%)	1 (2%)	19 (33%)^a^	1 (2%)	21 (36%)
**Subgroup analysis based on specific observational designs**
RCTs vs. observational studies (*n = *32)	29 (91%)	6 (19%)	13 (41%)	3 (9%)	22 (69%)	1 (3%)	8 (25%)	1 (3%)	10 (31%)
RCTs vs. cohort studies (*n = *16)	16 (100%)	3 (19%)	8 (50%)	0	11 (69%)	0	5 (31%)	0	5 (31%)
RCTs vs. case-control studies (*n = *10)	9 (90%)	2 (20%)	2 (20%)	0	4 (40%	0	6 (60%)	0	6 (60%)

a Eight studies showed increased risk with RCTs; 11 studies showed increased risk with observational data.

#### Agreement and disagreement of results

In most of the methodological evaluations the results of the treatment effect agreed between types of study design [6],[116],[118],[120],[121],[123]–[131]. Most studies that showed agreement between study designs did not find a significant increase or significant decrease in the adverse effects under investigation (Table 1).

There were major discrepancies in one pooled set of results. Col et al. [119] found an increase in breast cancer with menopausal hormone therapy in RCTs but a decrease in observational studies.

There were other instances where although the direction of the effect was not in opposing directions, apparently different conclusions may have been reached had a review been restricted to either RCTs or observational studies, and undue emphasis was placed on statistical significance tests. For instance, a significant increase in an adverse effect could be identified in an analysis of RCT data, yet pooling the observational studies may have identified no significant difference in adverse effects between the treatment and control group. Table 1 shows that the most common discrepancy between study types occurred when one set of studies identified a significant increase whilst another study design found no statistically significant difference. Given the imprecision in deriving estimates of rare events, this may not reflect any real difference between the estimates from RCTs and observational studies, and it would be more sensible to concentrate on the overlap of CIs rather than the variation in size of the *p*-values from significance testing.

#### Ratio of risk ratio or odds ratios estimates

RRs or ORs from the RCTs were compared to those from the observational studies by meta-analysis of the respective ROR for each adverse effect.

#### RCTs versus all “observational” studies

**Figure 2:**
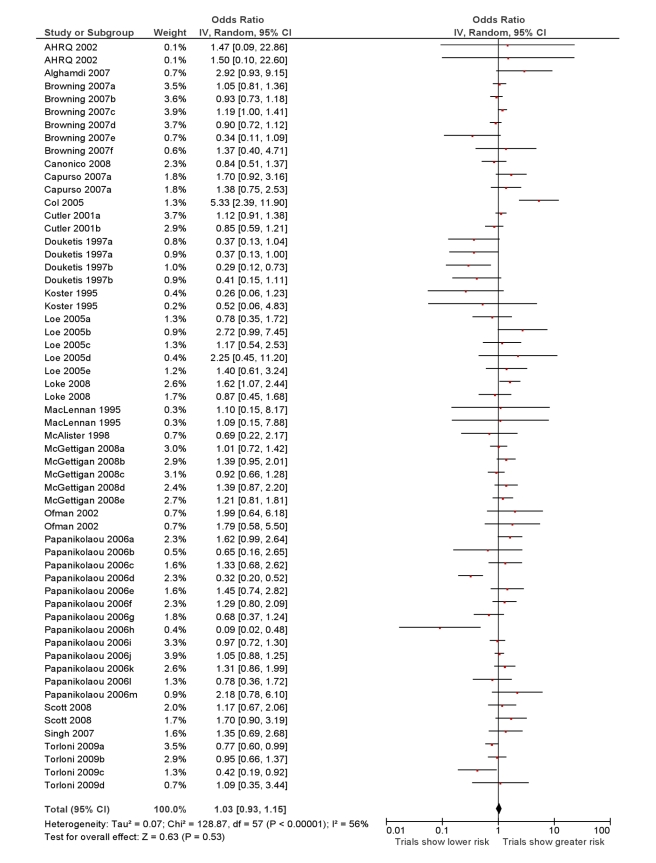
Meta-analysis of RORs from RCTs versus all observational studies. Studies are listed by first author's last name and year of publication (Loke 2008 is [124]; AHRQ 2002 is [114]). In some studies more than one outcome or intervention was assessed. In these instances, indicated by the lowercase letters after the study year, the data were entered in the meta-analysis separately. Other studies compared RCTs to cohort studies and case-control studies separately and therefore are listed twice (with no lowercase letter after the study year).

The overall ROR from meta-analysis using the data from all the studies that compared RCTs with either cohort studies or case-control studies, or that grouped studies under the umbrella of “observational” studies was estimated to be 1.03 (95% CI 0.93–1.15) with moderate heterogeneity (*I* ^2^ = 56%, 95% CI 38%–67%) (Figure 2).

**Figure 3:**
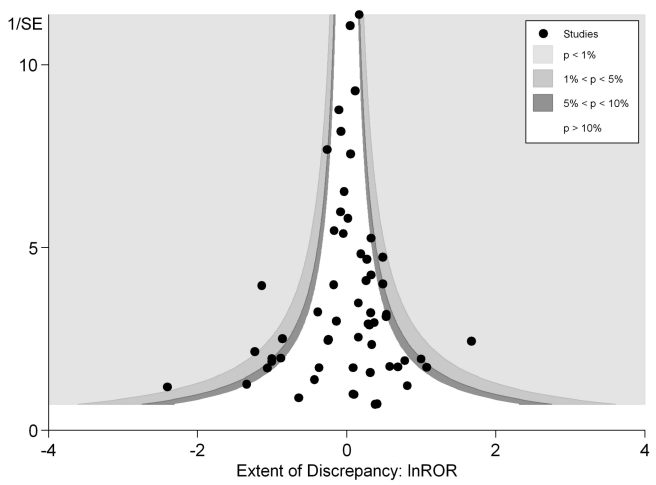
Contour funnel plot: discrepancy (ln ROR) between study designs in relation to precision of estimates (1/SE).

In Figure 3 we plotted the magnitude of discrepancy (ROR) from each meta-analysis against the precision of its estimates (1/SE), with the contour lines showing the extent of statistical significance for the discrepancy. Values on the *x*-axis show the magnitude of discrepancy, with the central ln ROR of zero indicating no discrepancy, or complete agreement between the pooled OR estimated from RCTs and observational studies. The *y*-axis illustrates the precision of the estimates (1/SE), with the data points at the top end having greater precision. This symmetrical distribution of the RORs of the various meta-analyses around the central ln ROR value of zero illustrates that random variation may be an important factor accounting for discrepant findings between meta-analyses of RCTs versus observational studies. If there had been any systematic and consistent bias that drove the results in a particular direction for certain study designs, the plot of RORs would likely be asymmetrical. The vertically tapering shape of the funnel also suggests that the discrepancies between RCTs and observational studies are less apparent when the estimates have greater precision. This may support the need for larger studies to assess adverse effects, whether they are RCTs or observational studies.

Both figures can be interpreted as demonstrating that there are no consistent systematic variations in pooled risk estimates of adverse effects from RCTs versus observational studies.

#### Sensitivity analysis: limiting to one review per adverse effect examined

There are no adverse effects for which two or more separate meta-analyses have used exactly the same primary studies (i.e., had complete overlap of RCTs and observational studies) to generate the pooled estimates. This reflects the different time periods, search strategies, and inclusion and exclusion criteria that have been used by authors of these meta-analyses such that even though they were looking at the same adverse effect, they used data from different studies in generating pooled overall estimates. As it turns out, the only adverse effect that was evaluated in more than one review was venous thromboembolism (VTE). There was some, but not complete, overlap of primary studies in three separate reviews of VTE with HRT (involving three overlapping case-control studies from a total of 18 observational studies analysed) and two separate reviews of VTE with oral contraceptives (one overlapping RCT, six [of 13] overlapping cohort studies, and two [of 20] overlapping case-control studies).

For the sensitivity analysis, we removed the three older meta-analyses pertaining to VTE so that the modest overlap could be further reduced, with only one review per specific adverse effect for the sensitivity analysis. The most recent meta-analyses for VTE (Canonico et al. [117] for VTE with HRT, Douketis et al. [121] for VTE with oral contraceptives) were used for analysis of the RORs. This yields RORs that are very similar to the original estimates: 1.06 (95% CI 0.96–1.18) for the overall analysis RCTs versus all observational studies, 1.00 (95% CI 0.71–1.42) for RCTs versus case-control studies and 1.07 (95% CI 0.86–1.34) for RCTs versus cohort studies.

#### Subgroup analysis

Subgroup analysis for comparison of RCTs against specific types of “observational” studies was carried out and is summarised in Table 2. Forest plots for each of these comparisons can be viewed in Figure S1.

**Table 2:** RORs from RCTs versus cohort studies, case-control studies, and studies described as “observational”.

Study Design Comparison	Pooled ROR (95% CI)	Heterogeneity
RCTs versus cohort studies	1.02 (0.82–1.28)	*I* ^2^ * = *43%
RCTs versus case-control studies	0.84 (0.57–1.23)	*I* ^2^ * = *54%
RCTs versus studies described as “observational”	1.08 (0.94–1.22)	*I* ^2^ * = *60%

## Discussion

Our analyses found little evidence of systematic differences in adverse effect estimates obtained from meta-analysis of RCTs and from meta-analysis of observational studies. Figure 3 shows that discrepancies may arise not just from differences in study design or systematic bias, but possibly because of the random variation, fluctuations or noise, and imprecision in attempting to derive estimates of rare events. There was less discrepancy between the study designs in meta-analyses that generated more precise estimates from larger studies, either because of better quality, or because the populations were more similar (perhaps because large, long-term RCTs capture a broad population similar to observational studies). Indeed, the adverse effects with discrepant results between RCTs and observational studies were distributed symmetrically to the right and left of the line of no difference, meaning that neither study design consistently over- or underestimates risk of harm as compared to the other. It is likely that other important factors such as population and delivery of intervention are at play here—for instance, the major discrepancy identified in Col et al. [119] for HRT and breast cancer is already well documented. This discrepancy has also been explained by the timing of the start of treatment relative to menopause, which was different between trials and observational studies. After adjustment, the results from the different study designs have been found to no longer differ [132],[133].

Most of the pooled results from the different study designs concurred in terms of identifying a significant increase or decrease, or no significant difference in risk of adverse effects. On the occasions where a discrepancy was found, the difference usually arose from a finding of no significant risk of adverse effects with one study design, in contrast to a significant increase in adverse effects from the other study design. This may reflect the limited size of the included studies to identify significant differences in rare adverse effects.

The increased risk in adverse effects in some studies was not consistently related to any particular study design—RCTs found a significant risk of adverse effects associated with the intervention under investigation in eight instances, while observational studies showed a significantly elevated risk in 11 cases.

Although reasons for discrepancies are unclear, specific factors which may have led to differences in adverse effect estimates were discussed by the respective authors. The differences between observational studies and RCTs in McGettigan and Henry's meta-analysis of cardiovascular risk were thought to be attributable to different dosages of anti-inflammatory drugs used [127]. Differences in Papanikolaou et al. [6] and Col et al. [119] were attributed to differing study populations. Other methodological evaluations discussed the nature of the study designs themselves being a factor that may have led to differences in estimates. For example, some stated that RCTs may record a higher incidence of adverse effects because of closer monitoring of patients, longer duration of treatment and follow-up, and more thorough recording, in line with regulatory requirements [6],[128]. Where RCTs had a lower incidence of adverse effects, it was suggested that this could be attributed to the exclusion of high-risk patients [119] and possibly linked to support by manufacturers [6].

The overall ROR did not suggest any consistent differences in adverse effects estimates from meta-analysis of RCTs versus meta-analysis of observational studies. This interpretation is supported by the funnel plot in Figure 3, which shows that differences between the results of the two study designs are equally distributed across the range. Some discrepancies may arise by chance, or through lack of precision from limited sample size for detecting rare adverse effects. While there are a few instances of sizeable discrepancies, the pooled estimates in Figure 2 and Table 2 indicate that in the scheme of things (particularly where larger, more precise primary studies are available), meta-analysis of observational studies yield adverse effects estimates that broadly match those from meta-analysis of RCTs.

### Limitations

This systematic review of reviews and methodological evaluations has a number of limitations. When comparing the pooled results from different study designs it is important to consider any confounding factors that may account for any differences identified. For instance, if one set of studies was carried out on a younger cohort of patients, with a lower drug dosage, or with shorter duration of use, or relied on passive ascertainment of adverse effects data [6],[17],[52],[134], it might be expected that the magnitude of any adverse effects recorded would be lower. However, most of the methodological evaluations were not conducted with the primary aim of assessing differences in study design, but were systematic reviews with some secondary comparative evaluation of study design embedded.

Another constraint of our overview is that we accepted information and data as reported by the authors of the included meta-analyses. We did not attempt to source the primary studies contained in each meta-analysis, as this would have required extracting data from more than 550 papers. For instance, we relied on the authors' categorisation of study design but are aware that authors may not all have used the same definitions. This is a particular problem with observational studies, where it is often difficult to determine the methodology used in the primary study and categorise it appropriately. In order to overcome this limitation, we chose to base our analysis on RCTs compared to “all” observational studies (either cohort studies, case-control studies, or “observational” studies as defined by the author), with a subgroup analysis based on different types of observational designs.

Another important limitation to this review is the potentially unrepresentative sample used. Systematic reviews with embedded data comparing different study designs may have been missed. The search strategy used was limited to a literature search to identify methodological papers whose primary aim was to assess the influence of study design on adverse effects and to a sift of the full text of systematic reviews of adverse effects (as a primary outcome) from the Cochrane Database of Systematic Reviews and DARE. Nevertheless, it should be noted that the Cochrane Database of Systematic Reviews and DARE databases cover a large proportion of all systematic reviews and that systematic reviews in which adverse effects are included as a secondary aim are unlikely to present subgroup analysis by study design for the adverse effects data.

There was considerable heterogeneity between the comparisons of different studies, suggesting that any differences may be specific to particular types of interventions or adverse effects. It may be that particular types of adverse effects can be identified more easily via particular types of study designs [3],[14],[135],[136]. However, it was difficult to assess the methodological evaluations by type of adverse effects. This would be of interest, given that the literature suggests that RCTs may be better at identifying some types of adverse effects (such as common, anticipated, and short-term) than observational studies.

### Future Research

Where no randomized data exist, observational studies may be the only recourse [137]. However, the potential value of observational data needs to be further demonstrated, particularly in specific situations where existing RCTs are short-term or based on highly selected populations. Comparisons of risk estimates from different types of observational studies (e.g., case-control as opposed to cohort) merit further assessment.

In addition, it would be useful (based on a case-control type of design) to carry out an in-depth examination of the meta-analyses (and their included primary studies) with substantial discrepancy amongst the RCTs and observational studies, as compared to other meta-analyses where RCTs and observational studies had close agreement. Any future research in this area should look into the role of confounding factors (such as different population selection and duration of drug exposure) between studies, and lack of precision in point estimates of risk for rare events that could have accounted for discrepant findings amongst RCTs and observational studies.

### Conclusions

Our findings have important implications for the conduct of systematic reviews of harm, particularly with regards to selection of a broad range of relevant studies. Although there are strengths and weaknesses to each study design, empirical evidence from this overview indicates that there is no difference on average between estimates of the risk of adverse effects from meta-analyses of RCTs and of observational studies. Instead of restricting the analysis to certain study designs, it may be preferable for systematic reviewers of adverse effects to evaluate a broad range of studies that can help build a complete picture of any potential harm and improve the generalisability of the review without loss of validity.

## Supporting Information

Figure S1 **Meta-analysis of RORs from RCTs versus cohort studies, case-control studies and studies described as “observational.”**
(DOC)

Table S1 **Characteristics of included studies.**
(DOC)

Text S1 **Sources searched for included studies.**
(PDF)

Text S2 **Example search strategy.**
(PDF)

Text S3 **Excluded studies.**
(PDF)

Text S4 **Duplicate data decisions.**
(PDF)

## Abbreviations

CI: confidence interval
DARE: Database of Abstracts of Reviews of Effects
HRT: hormone replacement therapy
OR: odds ratio
RCT: randomised controlled trial
ROR: ratio of odds ratios
RR: risk ratio
SE: standard error
VTE: venous thromboembolism
