# Supplementary material for: Meta-analyses of Adverse Effects Data Derived from Randomised Controlled Trials as Compared to Observational Studies: Methodological Overview
Source: PLoS Med. 2011 May 3;8(5):e1001026. doi: 10.1371/journal.pmed.1001026 (PMC3086872; doi:10.1371/journal.pmed.1001026)
Supplement: Figure S1 — Meta-analysis of RORs from RCTs versus cohort studies, case-control studies and studies described as “observational.” (DOC) [file pmed.1001026.s001.doc]

**Figure S1. Meta-Analysis of RORs from** **RCTs versus cohort studies, case control studies and studies described as ‘observational’**

**---------------------------------------------------------------------------------------**

**Figure 1: Meta-analysis of RORs from RCTs versus cohort studies**

**Figure 2: Meta-analysis of RORs from RCTs versus case-control studies**

**Figure 3: Meta-analysis of RORs from RCTs versus studies described as ‘observational’**

**Key to Study Outcomes**

In some studies more than one outcome was assessed. In these instances the data were entered in the meta-analysis separately;

Browning 2007a Breast cancer

Browning 2007b Prostate cancer

Browning 2007c Colorectal cancer

Browning 2007d Lung cancer

Browning 2007e Melanoma

Browning 2007f Gastric cancer

Cutler 2001a Acute graft

Cutler 2001b Chronic graft

Douketis 1997a Oral contraceptives

Douketis 1997b Hormone replacement therapy

Loe 2005a Intraventricular hemorrhage

Loe 2005b Bronchopulmonary dysplasia

Loe 2005c Patent ductus arteriosus

Loe 2005d Necrotizing enterocolitis

Loe 2005e Mortality

McGettigan 2008a Rofecoxib

McGettigan 2008b Celecoxib

McGettigan 2008c Naproxen

McGettigan 2008d Ibuprofen

McGettigan 2008e Diclofenac

Papanikolaou 2006a Convulsions with pertussis vaccine

Papanikolaou 2006b Hypotonic hyporesponsiveness with pertussis vaccine

Papanikolaou 2006c Major extracranial bleed with oral anitcoagulant therapy

Papanikolaou 2006d Symptomatic intracranial bleed with anticoagulant versus antiplatelet

Papanikolaou 2006e Major extracranial bleed with anticoagulant versus antiplatelet

Papanikolaou 2006f Major extracranial bleed with antiplatelet therapy

Papanikolaou 2006g Symptomatic intracranial bleed with antiplatelet therapy

Papanikolaou 2006h Visceral or vascular injury with labaroscopy versus open surgey for inguinal hernia

Papanikolaou 2006i Wound infection with laparoscopy versus open surgery for appendicitis

Papanikolaou 2006j Spontaneous miscarriage with folate supplementation

Papanikolaou 2006k Multiple gestation with folate supplementation

Papanikolaou 2006l Major bleed with platelet glycoprotein IIB/IIIA blocker therapy in PCI

Papanikolaou 2006m Acute myocardial infarction with rofecoxib versus naproxen therapy

Torloni 2009a Low birth weight

Torloni 2009b Dyslexia

Torloni 2009c Impaired hearing
