# Supplementary material for: Meta-analyses of Adverse Effects Data Derived from Randomised Controlled Trials as Compared to Observational Studies: Methodological Overview
Source: PLoS Med. 2011 May 3;8(5):e1001026. doi: 10.1371/journal.pmed.1001026 (PMC3086872; doi:10.1371/journal.pmed.1001026)
Supplement: Table S1 — Characteristics of included studies. (DOC) [file pmed.1001026.s002.doc]

**Table S1.** Characteristics of included studies

| **Reference** | **Study Design** | **Included Studies** | **Methodological Assessment** | **Increase/decrease/no difference in adverse effects by study design** |
| --- | --- | --- | --- | --- |
| **Agency for Healthcare Research and Quality 2002 [103]** | Systematic review of hormone replacement therapy and venous thromboembolism. | ***Venous thromboembolism***  **3 RCTs (N=3842)**  RR 3.08 (0.21-45.14)  **1 cohort study (N=112593)**  RR 2.1 (1.2-3.8)  **8 case-control studies (N=23544)**  RR 2.05 (1.40-2.95) | **Confounding factors by study design:** NR  **Heterogeneity within study designs:** NR (No significant heterogeneity among all 12 studies P>0.10)  **Statistical analysis comparing study designs:** NR | ***Venous thromboembolism***  **RCTs:** No significant difference  **Cohort study:** Significant increase  **Case-control studies:** Significant increase  **CI overlap:** Yes |
| **Alghamdi et al 2007 [104]** | Systematic review of preoperative aspirin and bleeding. | ***Rexploration***  **4 RCTs (N=1002)**  Aspirin 41/588 Contol 7/420  RR 3.71 (1.74-7.91)  Chi^2^=1.00, df=3 P=0.80, I^2^=0%  **5 Cohort studies (N=716)**  Aspirin 10/311 Control 11/405  RR 1.27 (0.54-2.98)  Chi^2^=1.95, df=3 P=0.58, I^2^=0% | **Confounding factors by study design:** NR but carries out sensitivity analysis by era (before 1990 and after) which suggests effect size larger in early era studies. RCTs tended to be earlier era studies but no further analysis conducted.  **Heterogeneity within study designs:**  No significant heterogeneity: one set of RCTs, one set of cohort studies  **Statistical analysis comparing study designs:** NR but no significant heterogeneity when all RCTs and cohort studies are pooled. | ***Reexploration***  **RCTs**: Significant increase P<0.001  **Cohort studies:** No significant difference P=0.58  **CI overlap:** Yes |
| **Browning and Martin 2007 [105]** | Systematic review of statins and cancer. | ***Breast cancer***  **7 RCTs (N=60917)**  RR 1.01 (0.79-1.30)  I^2^=43%  **9 Observational studies (N=688052)**  RR 0.96 (0.90-1.04)  I^2^=0%  ***Prostate cancer***  **4 RCTs (N=21740)**  RR 1.00 (0.85-1.17)  I^2^=0%  **5 Observational studies (N=375290)**  RR 1.08 (0.91-1.30)  I^2^=77%  ***Colorectal cancer***  **9 RCTs (N=67656)**  RR 1.02 (0.89-1.16)  I^2^=0%  **5 Observational studies (N=508696)**  RR 0.86 (0.77-0.96)  I^2^=89%  ***Lung cancer***  **9 RCTs (N=69301)**  RR 0.96 (0.84-1.09)  I^2^=0%  **3 Observational studies (N=372592)**  RR 1.07 (0.89-1.28)  I^2^=0%  ***Melanoma***  **4 RCTs (N=24222)**  RR 0.86 (0.62-1.20)  I^2^=17%  **1 Observational study (N=18047)**  RR 2.50 (0.83-7.55)  I^2^=NA  ***Gastric cancer***  **1 RCT (N=4444)**  RR 1.00 (0.35-2.85)  I^2^=NA  **2 Observational studies (N=37838)**  RR 0.73 (0.38-1.40)  I^2^=0% | **Confounding factors by study design:**  NR but acknowledges that counfounding and other bias may have had an effect  **Heterogeneity within study designs:**  No significant heterogeneity: Five sets of RCTs and three sets of observational studies  Signifiant heterogeneity: Two sets of observational studies  **Statistical analysis comparing study designs:** NR | ***Breast cancer***  **RCTs:** No significant difference P=0.92  **Observational studies:** No significant difference P=0.31  **CI overlap:** Yes  ***Prostate cancer***  **RCTs:** No significant difference P=0.99  **Observational studies:** No significant difference P=0.38  **CI overlap:** Yes  ***Colorectal cancer***  **RCTs:** No significant difference P=0.83  **Observational studies:** Significant decrease P=0.009  **CI overlap:** Yes  ***Lung cancer***  **RCTs:** No significant difference P=0.49  **Observational studies:** No significant difference P=0.50  **CI overlap:** Yes  ***Melanoma***  **RCTs:** No significant difference P=0.38  **Observational studies:** No significant difference P=0.10  **CI overlap:** Yes  ***Gastric cancer***  **RCTs:** No significant difference P=0.99  **Observational studies:** No significant difference P=0.34  **CI overlap:** Yes |
| **Canonico et al 2008 [106]** | Systematic review of HRT and venous thromboembolism | ***Venous thromboembolism***  **9 RCTs (N=NR)**  HRT 311/NR Placebo 146/NR  OR 2.1 (1.4-3.1)  Chi^2^ P=0.03, I^2^=58.9%  **8 observational studies (N=NR)**  OR 2.5 (1.9-3.4)  Chi^2^  P=0.03, I^2^=53.3% | **Confounding factors by study design:**  Acknowledges that ‘This difference could be explained by inclusion of procedure related venous thromboembolism in the women’s health initiative trials as well as the high degree of non-adherence to study drugs in the randomised controlled trials, resultin in an underestimation of hormone effects in the randomised controlled trials’.  **Heterogeneity within study designs:**  Significant heterogeneity: One set RCTs and one set of observational studies  **Statistical analysis comparing study designs:** NR | ***Venous thromboembolism***  **RCTs:** Significant increase  **Observational Studies:** Significant increase  **CI overlap:** Yes |
| **Capurso et al 2007 [107]** | Systematic review of NSAIDS and pancreatic cancer. | ***Pancreatic cancer (low NSAID exposure)***  **1 RCT (N=39,876)**  Aspirin/NSAIDS 30/19,934 Control 21/19,942  OR 1.43 (0.82-2.50)  **3 Cohort studies (N=1,072,263)**  Aspirin/NSAIDS 883/244,404 Control 3668/827,859  OR 0.84 (0.64-1.09)  **3 Case-control studies (N=7,254)**  Aspirin/NSAIDS 347/3,302 Control 728/3,952  OR 1.04 (0.81-1.33)  ***Pancreatic cancer (intermediate NSAID exposure)***  **3 Cohort studies (N=906,924)**  Aspirin/NSAIDS 363/79,065 Control 3,668/827,859  OR 0.94 (0.63-1.40)  **3 Case-control studies (N=4,648)**  Aspirin/NSAIDS 123/696 Control 728/3,952  OR 1.15 (0.69-1.91)  ***Pancreatic cancer (high NSAID exposure)***  **3 Cohort studies (N=851,932)**  Aspirin/NSAIDS 84/24,073 Control 3668/827,859  OR 0.94 (0.51-1.71)  **3 Case-control studies (N=4,267)**  Aspirin/NSAIDS 60/315 Control 728/3,952  OR 1.12 (0.52-2.41) | **Confounding factors by study design:** NR but conducts subgroup analysis by factors such as gender, aspirin use only, and nurse occupation.  **Heterogeneity within study designs:** NR (Significant heterogeneity among 7 studies with low exposure P=0.005, I^2^ =67.3%, 6 studies with intermediate exposure P=0.001, I^2^=75.0% and 6 studies with high exposure P<0.0001, I^2^=83.4%)  **Statistical analysis comparing study designs:** NR but states no significant difference. | ***Low exposure***  **RCT:** No significant difference  **Cohort studies:** No significant difference  **Case-control studies:** No significant difference  **CI overlap:** Yes  ***Intermediate exposure***  **Cohort studies:** No significant difference  **Case-control studies:** No significant difference  **CI overlap:** Yes  ***High exposure***  **Cohort studies:** No significant difference  **Case-control studies:** No significant difference  **CI overlap:** Yes |
| **Col et al 2005 [108]** | Systematic review of menopausal hormone therapy and breast cancer. | ***Breast cancer***  **2 RCTs (N=445)**  RR 3.41 (1.59-7.33)  Q=0.25  **8 Observational studies (N=3710)**  RR 0.64 (0.50-0.82)  Q=7.18 | **Confounding factors by study design:** Acknowledges possible confounding factors such as younger populationwith more favourable prognoistic profiles in observational studies. Also commented that observational studies lacked proper design and were more like reports of clinical experiences.  **Heterogeneity within study designs:** No significant heterogeneity: one set of RCTs and one set of observational studies  **Statistical analysis comparing study designs:** Significant heterogeneity exists if RCT and observational data are pooled. | ***Breast cancer***  **RCTs:** Significant increase P=0.0016  **Observational studies:**  Significant decrease P=0.00041  **CI overlap:** No |
| Cutler et al 2001 [109] | Systematic review of allogeneic peripheral -blood stem-cell and bone marrow transplantation and acute and chronic graft-versus-host disease | ***Transplantation (acute graft)***  **5 RCTs (N=699)**  RR 1.23 (1.05-1.45)  **10 Cohort studies (N=1371)**  RR 1.10 (0.96-1.26)  ***Transplantation (chronic graft )***  **5 RCTs (N=699)**  RR 1.37 (1.08-1.74)  **9 Cohort studies (N=1364)**  RR 1.62 (1.24-2.12) | **Confounding factors by study design:** NR  **Heterogeneity within study designs:** NR  **Statistical analysis comparing study designs:** NR | ***Acute graft***  **RCTs: S**ignificant increase **Cohort studies:** No significant difference  **CI overlap:** yes  ***Chronic graft***  **RCTs: S**ignificant increase P=0.01  **Cohort studies: S**ignificant increase P<0.001  **CI overlap:** yes |
| **Douketis et al 1997 [110]** | Systematic review of oral contraceptives and hormone replacement therapy and venous thromboembolism. | ***Venous thromboembolism (oral contraceptives)***  **1 RCT (N=NR)**  RR 1.1 (0.4-2.9)  **7 cohort studies****  RR 3.0 (2.2-4.2)  prospective studies Chi^2^ P=0.8, retrospective studies Chi^2^ P=0.3  **12 case-control studies (N=NR)**  RR 3.0 (2.6-3.4)  Chi^2^ P=<0.001  ***Venous thromboembolism (hormone replacement therapy)***  **2 RCTs (N=NR)**  RR 0.7 (0.3-1.6)  Chi^2^ P=0.72  **1 cohort study (N=NR)**  RR 1.7 (1.0-2.9)  **5 case-control studies (N=NR)**  RR 2.4 (1.7-3.5)  Chi^2^ P=0.24 | **Confounding factors by study design:**  NR  **Heterogeneity within study designs:**  No significant heterogeneity: one set of RCTs, one set of case-control studies, (one set of prospective cohort studies  P=0.8 and one set of retrospective cohort studies P=0.3)  Significant heterogeneity: One set of case-control studies  **Statistical analysis comparing study designs:** NR | ***Oral contraceptives***  **RCT:** No significant difference  **Cohort studies:** Significant increase  **Case-control studies:** Significant increase  **CI overlap:** Yes  ***Hormone replacement therapy***  **RCTs:** No significant difference  **Cohort studies:**  No significant difference  **Case-control studies:** Significant increase  **CI overlap:** No, RCTs have a lower risk ratio than case-contol studies. |
| **Koster et al 1995 [111]** | Systematic review of oral contraceptives and venous thromboembolism. | ***Venous thromboembolism***  **1 RCT (N=NR)**  RR 1.1 (0.4-2.9)  **6 Cohort studies (N=NR)**  RR 2.1 (0.3-16)  **8 Case-control studies (N=NR**)  RR 4.2 (1.3-14) | **Confounding factors by study design:**  NR but author states differences may be due to study bias  **Heterogeneity within study designs:** NR (Significant heterogeneity among all studies P<0.001)  **Statistical analysis comparing study designs:** NR | ***Venous thromboembolism***  **RCT:** No significant difference  **Cohort studies:** No significant difference  **Case-control studies:**  Significant increase  **CI overlap:** Yes |
| **Loe et al 2005 [112]** | Systematic review of neonatal safety and indomethacin tocolysis (an NSAID). | ***Intraventricular hemorrhage***  **9 RCTs (N=533)**  Study Group 22/263 Comparison group 23/270  OR 1.02 (0.55-1.89)  Heterogenity: P=0.93  **10 Observational studies (N=1241)**  Study Group 134/572 Comparison group 126/669  OR 1.31 (0.79-2.15)  Heterogenity: P=0.01  ***Bronchopulmonary dysplasia***  **3 RCTs (N=156)**  Study Group 15/76 Comparison group 6/80  OR 2.80 (1.07-7.31)  Heterogenity: P=0.12  **9 Observational studies (N=998)**  Study Group 118/451 Comparison group 136/547  OR 1.03 (0.76-1.40)  Heterogenity: P=0.49  ***Patent ductus arteriosus***  **6 RCTs (N=308)**  Study Group 21/153 Comparison group 18/155  OR 1.25 (0.64-2.54)  Heterogenity: P=0.78  **11 Observational studies (N=1948)**  Study Group 179/563 Comparison group 368/1385  OR 1.07 (0.76-1.52)  Heterogenity: P=0.05  ***Necrotizing enterocolitis***  **6 RCTs (N=329)**  Study Group 7/162 Comparison group 2/167  OR 2.43 (0.73-8.03)  Heterogenity: P=0.95  **11 Observational studies (N=2725)**  Study Group 157/956 Comparison group 385/1769  OR 1.08 (0.37-3.13)  Heterogenity: P=0.00  ***Mortality***  **9 RCTs (N=572)**  Study Group 15/283 Comparison group 11/289  OR 1.39 (0.65-2.97)  Heterogenity: P=0.99  **9 Observational studies (N=1234)**  Study Group 69/547 Comparison group 84/687  OR 0.99 (0.70-1.40)  Heterogenity: P=0.21 | **Confounding factors by study design:**  Meta-regression analysis with study location, study year, and presence or absence of tocolytics as covariates did not alter the results for RCTs or observational studies. Acknowledges that discrepancies may be due to differences in interventions, population, clinical studies and follow-up, as well as confounding / selection bias in non-randomised study designs.  **Heterogeneity within study designs:** No significant heterogeneity: Five sets of RCTs and two sets of observational studies  Significant heterogeneity: Three sets of observational studies  **Statistical analysis comparing study designs:** NR | ***Intraventricular hemorrhage***  **RCTs:**  No significant difference  **Observational studies:**  No significant difference  **CI overlap:** yes  ***Bronchopulmonary dysplasia***  **RCTs:**  Significant increase  **Observational studies:**  No significant difference  **CI overlap:** yes  ***Patent ductus arteriosus***  **RCTs:**  No significant difference **Observational studies:**  No significant difference  **CI overlap:** yes  ***Necrotizing enterocolitis***  **RCTs:**  No significant difference  **Observational studies:**  No significant difference  **CI overlap:** yes  ***Mortality***  **RCTs:**  No significant difference  **Observational studies:**  No significant difference  **CI overlap:** yes |
| **Loke et al 2008 [113]** | Systematic review of thiazolidinediones and fractures | ***Fractures among women***  **5 RCTs (N=4400)**  Thiazolidinedione 111/1903 Control 76/2497  OR 2.23 (1.65-3.01)  I^2^=0%  **1 Cohort study**  OR 1.38 (1.03-1.82)  **1 Case-control study**  OR 2.56 (1.43-4.58) | **Confounding factors by study design:**  NR but authors acknowledge that the trials contained relatively young participants and the case-control study involved an older population.  **Heterogeneity within study designs:** No significant heterogeneity: one set of RCTs  **Statistical analysis comparing study designs:** NR | ***Fractures among women***  **RCTs**  Significant increase P<0.001  **Cohort study**  Significant increase  **Case-control study**  Significant increase  **CI overlap:** yes |
| **MacLennan et al 1995 [114]** | Systematic review of oestrogen replacement therapy and colorectal cancer. | ***Colorectal cancer***  **1 RCT (N=168)**  RR 1.0 (0.14-7.1)  **4 Cohort studies (N=169400)**  RR 0.91 (0.60-1.38)  Woolf’s test, P=0.89  **9 Case-control studies (N=8631)**  RR 0.92 (0.71-1.20)  Woolf’s test, P<0.01 | **Confounding factors by study design:**  Acknowledges insufficient information on dose duration to check variables.  **Heterogeneity within study designs:** No significant heterogeneity: one set of cohort studies.  Significant heterogeneity: one set of case-control studies.  **Statistical analysis comparing study designs:** NR | ***Colorectal cancer***  **RCT:**  No significant difference  **Cohort studies:**  No significant difference  **Case-control studies:**  No significant difference  **CI overlap:** Yes |
| **McAlister et al 1998 [115]** | Systematic review of perioperative allogeneic blood transfusion. | ***Mortality***  **5 RCTs (N=1923)**  Treatment 164/978 Control 169/945  RR 0.94 (0.76-1.16)  **1 Cohort Study (N=273)**  Treatment 5/94 Control 7/179  RR 1.36 (0.44-4.17) | **Confounding factors by study design:**  NR  **Heterogeneity within study designs:** NR (No indication of heterogeneity for all 6 studies P>0.45)  **Statistical analysis comparing study designs:** NR | ***Mortality***  **RCTs:**  No significant difference  **Cohort Studies:**  No significant difference  **CI overlap:** yes |
| **McGettigan and Henry 2008 [116]** | Compares the risk ratios of cardiovascular events from systematic reviews of RCTs and systematic reviews of observational studies. | ***CV events (Rofecoxib)***  **Review 1: 37 RCTs (N=13053)**  Treatment 98/6638 Control 72/6415  RR 1.38 (1.01-1.87)  **Review 3: 13 observational studies (N=NR)**  RR 1.36 (1.18-1.56)  Chi^2^ P<0.00001, I^2^=84.3%  ***CV events (Celecoxib)***  **Review 1: 41 RCTs (N=13929)**  RR 1.51 (1.02- 2.04)  Treatment 84/8976 Control29/4953  **Review 3: 13 observational studies (N=NR)**  RR 1.09 (0.95-1.25)  Chi^2^ P<0.00001 I^2^=86.7%  ***CV events (Naproxen)***  **Review 1: 42 RCTs (N=NR)**  RR 0.92 (0.67-1.26)  **Review 3: 15+ observational studies (N=NR)**  RR 1.0 (0.91-1.09)  ***CV events (Ibuprofen)***  **Review 1: 24 RCTs (N=NR)**  RR 1.51 (0.96-2.37)  **Review 3: 16+ observational studies (N=NR)**  RR 1.09 (0.99-1.20)  ***CV events (Diclofenac)***  **Review 1: 26 RCTs (N=NR)**  RR 1.63 (1.12-2.37)  **Review 3: 12 observational studies (N=NR)**  RR 1.35 (1.16-1.58)  Chi^2^ P<0.00001 I^2^=87.0% | **Confounding factors by study design:** Controlled for some study design factors including age, gender, dose, and type of drug, found that discrepant estimates for celecoxib and ibuprofen were due to higher doses of drugs used in RCTs than in observational studies (where reported).  **Heterogeneity within study designs:** Significant heterogeneity: Three sets of observational studies  **Statistical analysis comparing study designs:** NR | ***Rofecoxib***  **RCTs:** Significant increase  **Observational Studies:** Significant increase  **CI overlap:** yes  *C****elecoxib***  **RCTs:** Significant increase  **Observational Studies:** No significant difference  **CI overlap:** yes  ***Naproxen***  **RCTs:** No significant difference  **Observational Studies:** No significant difference  **CI overlap:** yes  ***Ibuprofen***  **RCTs:** No significant difference  **Observational Studies:** No significant difference  **CI overlap:** yes  ***Diclofenac***  **RCTs: S**ignificant increase  **Observational Studies:**  Significant increase  **CI overlap:** yes |
| **Ofman et al 2002[117]** | Systematic review of NSAIDs and severe upper gastrointestinal complications perforations, ulcers and bleeds. | ***Perforations, ulcers and bleeds***  **16 RCTs (N=4431)**  OR 5.36 (1.76-16.1)  **9 Cohort studies (N=758776 patient-years)**  RR 2.7 (2.1-3.5)  **23 Case-control studies (N=25732)**  OR 3.0 (2.5-3.7) | **Confounding factors by study design:**  NR but states that data were insufficient to justify subgroup analysis by age, comorbid conditions, drug or dose.  **Heterogeneity within study designs:** NR (Only pooled homogeneous studies for each study design)  **Statistical analysis comparing study designs:** NR | ***Perforations, ulcers and bleeds***  **RCTs:** Significant increase  **Cohort studies:** Significant increase  **Case-control studies:** Significant increase  **CI overlap:** Yes |
| **Papanikolaou et al 2006 [6]** | Compares evidence on 15 harms with drugs, vitamins, vaccines and surgical procedures in RCTs identified from Cochrane reviews and non-RCTs from MEDLINE. | ***Convulsions with pertussis vaccine***  **15 RCTs (N=124387)**  RR 0.47 (0.31-0.73)  **2 Non-RCTs (N=NR)**  RR 0.29 (0.23-0.37)  ***Hypotonic hyporesponsiveness with pertussis vaccine***  **11 RCTs (N=121573)**  RR 0.26 (0.08-0.81)  Q, P<0.10  **1 Non-RCT (N= NR)**  RR 0.40 (0.18-0.89)  ***Major extracranial bleed with oral anitcoagulant therapy***  **16 RCTs (N=22049)**  RR 3.31 (2.35-4.67)  **5 Non-RCTs (N=403397)**  RR 2.48 (1.39-4.44)  Q, P<0.10  ***Symptomatic intracranial bleed with anticoagulant versus antiplatelet***  **15 RCTs (N=22794)**  RR 2.64 (1.95-3.58)  **2 Non-RCTs (N=273722)**  RR 8.25 (5.58-12.18)  ***Major extracranial bleed with anticoagulant versus antiplatelet***  **6 RCTs (N=11721)**  RR 1.78 (0.93-3.40)  **1 Non-RCT (N=4249)**  RR 1.23 (1.05-1.44)  ***Major extracranial bleed with antiplatelet therapy***  **9 RCTs (N=41399)**  RR 1.68 (1.34-2.12)  **2 Non-RCTs (N=24966)**  RR 1.30 (0.85-1.97)  Q, P<0.10  ***Symptomatic intracranial bleed with antiplatelet therapy***  **9 RCTs (N=41399)**  RR 1.22 (1.00-1.50)  **2 Non-RCTs (N=36190)**  RR 1.80 (1.02-3.19)  Q, P<0.10  ***Visceral or vascular injury with labaroscopy versus open surgey for inguinal hernia***  **22 RCTs (N=4914)**  RR 1.56 (0.75-3.29)  **1 Non-RCT (N=5506)**  RR 17.30 (3.91-76.80)  ***Wound infection with laparoscopy versus open surgery for appendicitis***  **34 RCTs (N=4324)**  RR 0.56 (0.43-0.72)  **2 Non-RCTs (N=150017)**  RR 0.58 (0.50-0.68)  ***Spontaneous miscarriage with folate supplementation***  **3 RCTs (N=7600)**  RR 1.12 (0.98-1.29)  **2 Non-RCTs (N=20509)**  RR 1.07 (0.96-1.20)  ***Multiple gestation with folate supplementation***  **3 RCTs (N=6241)**  RR 1.40 (0.93-2.11)  **3 Non-RCTs (N=690395)**  RR 1.07 (0.98-1.17)  Q, P<0.10  ***Major bleed with platelet glycoprotein IIB/IIIA blocker therapy in PCI***  **12 RCTs (N=17469)**  RR 1.36 (1.04-1.77)  Q, P<0.10  **1 Non-RCT (N=18821)**  RR 1.74 (0.83-3.66)  ***Acute myocardial infarction with rofecoxib versus naproxen therapy***  **1 RCTs (N=8076)**  RR 2.86 (1.28-6.39)  **1 Non-RCT (N=90629)**  RR 1.31 (0.69-2.48)  Q, P<0.10 | **Confounding factors by study design:**  Acknowledges differences in populations between randomised and non-randomised studies.  **Heterogeneity within study designs:**  No significant heterogeneity: 12 sets of RCTs and 4 sets of non-RCTs  Significant heterogeneity: 2 sets of RCTs and 5 sets of non-RCTs.  **Statistical analysis comparing study designs:** Differences in risk ratio beyond chance between randomised and nonrandomised studies occurred for 2 of the 13 topics.  The estimated increase in risk ratio differed more than 2 fold in 7 of the 13 topics.  The estimated increase in risk differed more than 2 fold in 5 of the 8 topics. | ***Convulsions with pertussis vaccine***  **RCTs:** Significant decrease  **Non-RCTs:** Significant decrease  **CI overlap:** yes  ***Hypotonic hyporesponsiveness with pertussis vaccine***  **RCTs:** Significant decrease  **Non-RCTs:** Significant decrease  **CI overlap:** yes  ***Major extracranial bleed with oral anitcoagulant therapy***  **RCTs:** Significant increase  **NonRCTs:** Significant increase  **CI overlap:** yes  ***Symptomatic intracranial bleed with anticoagulant versus antiplatelet***  **RCTs:** Significant increase  **Non-RCTs:** Significant increase  **CI overlap:** no  ***Major extracranial bleed with anticoagulant versus antiplatelet***  **RCTs:** No significant difference  **Non-RCTs:** Significant increase  **CI overlap:** yes  ***Major extracranial bleed with antiplatelet therapy***  **RCTs:** Significant increase  **Non-RCTs:** No significant difference  **CI overlap:** yes  ***Symptomatic intracranial bleed with antiplatelet therapy***  **RCTs:** No significant difference  **Non-RCTs:** Significant increase  **CI overlap:** yes  ***Visceral or vascular injury with labaroscopy versus open surgey for inguinal hernia***  **RCTs:** No significant difference  **Non-RCTs:** Significant increase  **CI overlap:** no  ***Wound infection with laparoscopy versus open surgery for appendicitis***  **RCTs:** Significant decrease  **Non-RCTs:** Significant decrease  **CI overlap:** yes  ***Spontaneous miscarriage with folate supplementation***  **RCTs:** No significant difference  **Non-RCTs:** No significant difference  **CI overlap:** yes  ***Multiple gestation with folate supplementation***  **RCTs:** No significant difference  **Non-RCTs:** No significant difference  **CI overlap:** yes  ***Major bleed with platelet glycoprotein IIB/IIIA blocker therapy in PCI***  **RCTs:** Signigicant increase  **Non-RCTs:** No significant difference  **CI overlap:** yes  **Acute myocardial infarction with rofecoxib versus naproxen therapy**  **RCTs:** Significant increase  **Non-RCTs:** No significant difference  **CI overlap:** yes |
| **Scott et al 2008 [118]** | Systematic review of NSAIDS and cardiac failure. | ***Cardiac failure***  **6 RCTs (N=15750)**  NSAIDS 40/8542 Placebo 13/7208  OR 2.31 (1.34-4.00)  Chi^2^ P=0.37, I^2^=6.9%  **2 Cohort studies (82785 patient years)**  RR 1.97 (1.73-2.25)  Chi^2^ P=0.33, I^2^= 0%  **5 Case-control studies (N=50519)**  OR 1.36 (0.99-1.85)  Chi^2^ P<0.001, I^2^= 90.9% | **Confounding factors by study design:** NR but discusses the problems of over the counter NSAIDS in observational studies and that 2 RCTs and one case-control study excluded patients with previous cardiac failure. Also commented on short-term follow-up in RCTs,  **Heterogeneity within study designs:** No significant heterogeneity: one set of RCTs and one set of cohort studies.  Significant heterogeneity: one set of case-control studies.  **Statistical analysis comparing study designs:** NR | ***Cardiac failure***  **RCTs:** Significant increase  **Cohort studies:** Significant increase  **Case-control studies:** No significant difference  **CI overlap:** yes |
| **Singh et al 2007 [119]** | Systematic review of thiazolidinediones and heart failure. | ***Heart failure***  **3 RCTs (N=10731)**  Treatment 314/5350 Control 210/5381  OR 2.10 (1.08-4.08)  Chi^2^ P=0.09, I^2^=58.8%  **4 Observational studies (N=67382)**  OR 1.55 (1.33-1.80)  Chi^2^ P=0.13, I^2^=46.9% | **Confounding factors by study design:** NR  **Heterogeneity within study designs:** No significant heterogeneity: one set of observational studies  Signigicant heteregenetiy: one set of RCTs  **Statistical analysis comparing study designs:** NR | ***Heart failure***  **RCTs:** Significant increase P=0.03  **Observational studies:** Significant increase P<0.00001  **CI overlap:** yes |
| **Torloni et al 2009 [120]** | Systematic review of ultrasonography in pregnancy | ***Low birth weight***  **10 RCTs (N=24271)**  OR 1.06 (0.84-1.35)  I^2^=69.3%  **6 Cohort studies (N=18622)**  OR 1.11 (0.84-1.46)  I^2^=72.8%  **1 Case control study(N=12,546)**  1.38 (1.25-1.51)  ***Dyslexia***  **1 RCT (N=603)**  OR 0.75 (0.41-1.37)  **1 Cohort study (N=806)**  OR 1.78 (1.08-2.93)  ***Impaired hearing***  **1 RCT (N=2008)**  OR 0.97 (0.62-1.53)  **1 Cohort study (N=723)**  OR 0.89 (0.31-2.57) | **Confounding factors by study design:** NR  **Heterogeneity within study designs:**  No significant heterogeneity: one set of RCTs and one set of cohort studies. Significant heterogeneity: one set of RCTs and one set of cohort studies.  **Statistical analysis comparing study designs:** NR | ***Low birth weight***  **RCTs;** No significant difference  **Cohort studies:** No significant difference  **Case control study:** Significant increase  **CI overlap:** yes  ***Dyslexia***  **RCT:** No significant difference  **Cohort study:** Significant increase  **CI overlap:** yes  ***Impaired hearing***  **RCT:** No significant difference  **Cohort study:** No significant difference  **CI overlap:** yes |

**Key**

RR – Risk ratio

N – Number of study participants

WMD – Weighted Means Difference

OR – Odds Ratio

NR – Not reported

CI – Confidence Interval

**- data were calculated from information presented in paper
