# Supplementary material for: Meta-analyses of Adverse Effects Data Derived from Randomised Controlled Trials as Compared to Observational Studies: Methodological Overview
Source: PLoS Med. 2011 May 3;8(5):e1001026. doi: 10.1371/journal.pmed.1001026 (PMC3086872; doi:10.1371/journal.pmed.1001026)
Supplement: Text S1 — Sources searched for included studies. (PDF) [file pmed.1001026.s003.pdf]

Text S1. Sources searched for included studies

Databases
All databases were originally searched on the 26th or 27th September 2007 with the most recent update searches carried out between the 22nd October and 6th November 2009.

Cochrane Database of Systematic Reviews (CDSR): methodology reviews only: 2009 Issue 4
Cochrane Methodology Register (CMR): 2009 Issue 4
Database of Abstracts of Reviews of Effects (DARE): November 2009
EMBASE: 1980 to 2009 Week 42
Health Technology Assessment (HTA) Database: November 2009
Health Management Information Consortium (HMIC): September 2009
Index to Theses: November 2009
Library, Information Science & Technology Abstracts (LISTA): Mid-1960s – October 2009
MEDLINE: 1950 to October Week 3
MEDLINE in process: 22 October 2009

Handsearching of Journals
BMC Clinical Pharmacology - 2001;1(1) to 2009;9(17)
BMC Medical Research Methodology - 2001;1 to 2009;9(69)
Drug Safety – 1998;18(1) to 2009;32(11)
Health Information and Libraries Journal (formerly Health Libraries Review) - 1994;11(1) to 2009;26(3)
Journal of Clinical Epidemiology - 1998 to 2009;62(12)
Journal of Information Science - 1979;1(1) to 2009;35(5)
Journal of Librarianship and Information Science - 1969;1(1) to 2009;41(3)
Journal of the Medical Library Association (formerly the Bulletin of the Medical Library Association) - from 2000;88(2) to 2009;97(4)
Pharmacoepidemiology & Drug Safety – 1992;1(1) to 2009;18(11)

Handsearching of Conference Proceedings
Cochrane Colloquia 1994 - 2009
HTAi 2004 - 2009
Pharma-Bio-Med Conference and Exposition 2006 - 2008
Symposium on Systematic Reviews 1998 - 2002

Web Sources
Agency for Healthcare Research and Quality (AHRQ) via http://www.ahrq.gov/ Searched: 28/10/09
Health Technology Assessment Programme (HTA) via http://www.hta.ac.uk/index.shtml Searched: 28/10/09
