# Supplementary material for: Meta-analyses of Adverse Effects Data Derived from Randomised Controlled Trials as Compared to Observational Studies: Methodological Overview
Source: PLoS Med. 2011 May 3;8(5):e1001026. doi: 10.1371/journal.pmed.1001026 (PMC3086872; doi:10.1371/journal.pmed.1001026)
Supplement: Text S2 — Example search strategy. (PDF) [file pmed.1001026.s004.pdf]

Text S2. Example search strategy

Cochrane Methodology Register (CMR)
Interface: http://www.thecochrane library.com
Version: 2007 Issue 3 (original search), 2008 Issue 3 (first update search), 2009 Issue 4 (second update search)
Date Searched: 26/09/07 (original search), 17/08/09 (first update search), 22/10/09 (second update search)
The following search strategy, using terms in the title, abstract and keywords, retrieved 1517 records in the original search, an extra 249 records in the first update search and 344 in the second update search;

#1 adverse
#2 side next effect*
#3 unintended next effect*
#4 unintended next event*
#5 unintended next outcome*
#6 unintended next reaction*
#7 unintended next interaction*
#8 unintended next response*
#9 unintentional next effect*
#10 unintentional next event*
#11 unintentional next outcome*
#12 unintentional next reaction*
#13 unintentional next interaction*
#14 unintentional next response*
#15 unwanted next effect*
#16 unwanted next event*
#17 unwanted next outcome*
#18 unwanted next reaction*
#19 unwanted next interaction*
#20 unwanted next response*
#21 unexpected next effect*
#22 unexpected next event*
#23 unexpected next outcome*
#24 unexpected next reaction*
#25 unexpected next interaction*
#26 unexpected next response*
#27 undesirable next effect*
#28 undesirable next event*
#29 undesirable next outcome*
#30 undesirable next reaction*
#31 undesirable next interaction*
#32 undesirable next response*
#33 adrs or ades or adr
#34 drug next surveillance
#35 post next marketing next surveillance
#36 postmarketing next surveillance
#37 treatment next emergent
#38 complication*
#39 tolerability

#40 toxicity
#41 harm or harms or harmful
#42 safety
#43 safe
#44 tolerance
#45 tolerate
#46 toxic
#47 risk or risks
#48 (#1 OR #2 OR #3 OR #4 OR #5 OR #6 OR #7 OR #8 OR #9 OR #10 OR #11 OR #12 OR #13 OR #14 OR #15 OR #16 OR #17 OR #18 OR #19 OR #20 OR #21 OR #22 OR #23 OR #24 OR #25 or #26 or #27 or #28 or #29 or #30 or #31 or #32 or #33 or #34 or #35 or #36 or #37 or #38 or #39 or #40 or #41 or #42 or #43 or #44 or #45 or #46 or #47)
