# Supplementary material for: Meta-analyses of Adverse Effects Data Derived from Randomised Controlled Trials as Compared to Observational Studies: Methodological Overview
Source: PLoS Med. 2011 May 3;8(5):e1001026. doi: 10.1371/journal.pmed.1001026 (PMC3086872; doi:10.1371/journal.pmed.1001026)
Supplement: Text S3 — Excluded studies. (PDF) [file pmed.1001026.s005.pdf]

Text S3. Excluded Studies

67 studies were excluded from this systematic review. Nearly a third of these studies (20/68) did not compare a formally recognised study design (such as RCTs, cohort studies or case-control studies).{Blankensteijn, 2000 #9502; Choi, 2003 #9504; Collaborative Group on Hormonal Factors in Breast Cancer, 1996 #9549; Curran, 1999 #9556; Dezfulian, 2003 #9520; Eikelboom, 2001 #1280; García Rodríguez, 2001 #9501; Gordon, 2010 #11307; Hebert, 2007 #6329; Levine, 1997 #3187; Marang-van de Mheen, 2007 #8961; Marra, 2006 #9534; Martel, 2005 #9535; Molloy, 2002 #9503; Rothwell, 1996 #1368; Safdar, 2002 #9500; Vamvakas, 1995 #9511; Viboud, 2001 #6139; Glanz, 2006 #2695; van Staa, 2008 #6330} Thirteen studies were excluded because their hypothesis stated that the intervention had a protective (beneficial) effect.{Beral, 2008 #9529; Bonovas, 2007 #9528; Etminan, 2003 #9505; Fernandez, 2001 #9499; Hébert-Croteau, 1998 #9550; Kashyap, 2004 #9531; Larsson, 2006 #9532; Pladevall-Vila, 1996 #289; Reynolds, 2002 #9521; Toh, 2007 #9540; Wiens, 2006 #9541; Yaffe, 1998 #9551} 23 studies did not present the pooled data for each study design {Brumback, 1999 #26; Col, 2003 #369; Costa, 2006 #9524; Greiser, 2005 #9522; Grullon, 1997 #9492; Hall, 2006 #6323; Hawkey, 1990 #5470; Janowsky, 2000 #9498; Katerndahl, 1992 #9544; Kuoppala, 2008 #11302; Lawlor, 2003 #9519; Loke, 2009 #10572 ; Magee, 2001 #577; Martin, 2005 #2740; Miwa, 1997 #1465; Nakhai-Pour, 2008 #11289; Padwal, 2004 #9518; Rossi, 1983 #4958; Shah, 2005 #9538; Steffensmeier, 2006 #379; Steinberg, 1994 #157; Thavagnanam, 2007 #11300; Uboweja, 2006 #9543} 5 studies did not compare similar data, for example, similar adverse effects,{Demicheli, 2003 #9523; Ernst, 1998 #9494} similar comparators,{Egger, 1998 #9415} or similar outcome measures.{O'Brien, 2008 #11311; Singh, 2006 #2653} For three studies only the abstract was available. {Henry, 2001 #33; Vandermeer, 2004 #28; Zhang, 2001 #516} Two studies contained duplicate data from studies already included,{Miller, 2002 #5942; Meenan, 2002 #5937} one study did not include a health care intervention {Boyd, 2003 #9542} and lastly one study did not measure the magnitude or direction of any adverse effects but looked at the decline in risk.{Schaffer, 2006 #9537}
