# Supplementary material for: Meta-analyses of Adverse Effects Data Derived from Randomised Controlled Trials as Compared to Observational Studies: Methodological Overview
Source: PLoS Med. 2011 May 3;8(5):e1001026. doi: 10.1371/journal.pmed.1001026 (PMC3086872; doi:10.1371/journal.pmed.1001026)
Supplement: Text S4 — Duplicate data decisions. (PDF) [file pmed.1001026.s006.pdf]

Text S4. Duplicate data decisions

A major problem with the methodological evaluations was the issue of double data. Using the same data set more than once or multiple related outcomes in any analysis may make the data appear inappropriately homogenous and may present relationships which do not exist. The following decisions were, therefore, implemented before data entry into STATA.

1) Where both random effects models and fixed effects models were presented{Agency for Healthcare Research and Quality, 2002 #9553} the random effects models were selected over the fixed effects models as they represent a more conservative approach, and studies were often heterogeneous (where measured).
2) The separate analysis for each named cancer{Browning, 2007 #9527} were selected in preference to the grouped set of results of all cancers.
3) The systematic reviews with the largest number of included studies were selected from McGettigan and Henry 2008. {McGettigan, 2008 #6206}
4) Primary outcomes of fractures,{Loke, 2008 #10490} mortality, {McAlister, 1998 #9491} low birth weight,{Torloni, 2009 #11305} and dyslexia{Torloni, 2009 #11305} were selected in preference to the secondary outcomes of bone mineral density{Loke, 2008 #10490} cancer recurrence,{McAlister, 1998 #9491} preterm birth and other perinatal outcomes,{Torloni, 2009 #11305} and delayed speech.{Torloni, 2009 #11305}
5) Where outcomes were reported as risk ratios and weighted means difference, risk ratios were selected in preference to weighted means difference. Reexploration {Alghamdi, 2007 #9530} was therefore selected in preference to total blood loss{Alghamdi, 2007 #9530} or blood transfusions.
